# Some Aspects of Intestinal Obstruction

**Published:** 1908-05-30

**Authors:** 


### Points in Surgery.

### SOME ASPECTS OF INTESTINAL OBSTRUCTION.

### OBSTRUCTION OF THE BOWEL DUE TO COMPRESSION FROM WITHOUT.

Peritoneal adhesions are without doubt the commonest cause of constriction of the intestine from outside. They are in every case the outcome of an acute or sub-acute peritonitis which has subsided. Pelvic peritonitis associated with the genital organs in females and appendicitis in both sexes constitute the primary causes in most cases. The resulting adhesions connect some part of the intestine to the abdominal parietes or to other abdominal viscera or to adjacent coils of gut so that the affected coil is bent or distorted in such a manner that its lumen is appreciably narrowed. In the majority of cases the symptoms of obstruction do not make their appearance until months after the initial peritonitis.

Chronic Obstruction Due to Adhesions.

Peritoneal adhesions most often give rise to the acuter forms of obstruction, as, for example, when a coil of intestine is suddenly and tightly stretched across a band. It is less common for chronic obstruction to be produced in this way. When it does occur it is usually the result of two factors: (1) the bowel is bent to a moderate degree by adhesions which connect it to the parietes or to some other abdominal viscus, so that its lumen is narrowed without being obliterated; (2) the attachment of the adhesion to even a limited area of bowel wall causes an impediment to the passage of fæces, because the gut at the adherent spot cannot exercise its peristaltic action.

In other cases a single permanent loop in the bowel is formed by the adhesions, a condition which is often the result of inflammatory changes in the serous coat of a knuckle of gut which has descended into a hernia and been damaged there. In such a case a fold of mucous membrane projects into the lumen of the intestine, and offers a valve-like impediment to the passage of bowel contents. Or, again, coils of intestine may be found matted together in a confused rounded mass which compares conspicuously with the normal bowel. The coils in the mass are much distorted, and when obstruction has been caused, the coils entering the mass are seen to be dilated, while those leaving it are more or less shrunken. This condition follows on any localised form of peritonitis, but most commonly on the tuberculous variety.

The association of cicatricial strictures of the bowel with a constricting peritonitis is a well-recognised clinical condition. In fact, it must be the exception to find an ulcer of the bowel which is not accompanied by a local thickening of the serous coat in its neighbourhood. But Leichtenstern has also described a constriction of the large intestine by an annular peritoneal thickening where no ulcer is present. The condition is found at the flexures of the colon. According to him, the obstacles to the advance of bowel contents are greater in these situations, so that repeated fæcal accumulations are found there. Their presence sets up a chronic peritonitis which results in constriction. The probable explanation of these cases is that the localised subacute peritonitis is the outcome of a stercoral ulcer.

In all these cases the symptoms resemble those of intestinal obstruction from any other cause, and the diagnosis of the actual condition of affairs can only rest on the history of a past attack of peritoneal inflammation.

Compression from Other Causes.

Compression of the intestine apart from peritoneal adhesions is generally due to one of two causes—new growths or displaced viscera. It will be readily understood that those portions of the intestine which are fixed by their anatomical connections are the most liable to compression by such conditions. In fact, one might formulate it as a law that the liability of any portion of the intestine to undergo compression by a tumour or by a displaced organ is in inverse proportion to its mobility.

The rectum is especially prone to these accidents. It is not only fixed by its anatomical connections, but it is contained within the unyielding walls of the bony pelvic canal; and, moreover, it is in the female in intimate relation with the organs of generation, whose liability to displacement is greater than that of any other viscus. The great majority of these cases arise from compression of the rectum against the front of the sacrum by the body of the retroverted uterus. The bowel is unable to get out of the way and the fæces which collect above the obstruction press down on the fundus and tend to increase rather than diminish the gravity of the condition.

It might be thought that fibroid disease of the uterus would be among the commonest causes of compression of the rectum. But as a matter of practical experience, such is not the case. It is quite the exception for a fibromyoma to become impacted in Douglas' pouch and cause intestinal obstruction, and although this condition is mentioned in text-books of gynæcology as one of the indications for performing hysterectomy, it is rarely met with in actual practice.

On the other hand, the rectum is very prone to be compressed by malignant disease of the uterus or of the ovaries (carcinoma of which is quite common). If the disease spreads upwards the sigmoid or descending colon on the left side and the cæcum or ascending colon on the right side may be compressed. One very interesting case has been reported in which the cæcum was compressed by a tumour due to tubal pregnancy on the right side, and the abdomen was opened on account of symptoms of intestinal obstruction.

As might be expected from its peritoneal relations and its propinquity to the liver, the pancreas and the kidney, which are often the seat of malignant disease, the duodenum frequently suffers compression in this way. Conversely cases of compression of the transverse colon or of the small intestine are almost unknown.

In rare instances a retroperitoneal abscess such as a psoas abscess or a sarcoma of the posterior abdominal wall may compress the bowel. The symptoms presented vary in degree from those merely of troublesome constipation up to the syndrome which is characteristic of organic stenosis.

An absolute diagnosis of the cause is usually impossible, except in those cases where a rectal examination reveals a retroverted uterus pressing backwards on the rectum.

